# Current realities of home blood pressure monitoring from physicians’ perspectives: results from Asia HBPM survey 2020

**DOI:** 10.1038/s41440-023-01259-1

**Published:** 2023-04-11

**Authors:** Tzung-Dau Wang, Takayoshi Ohkubo, Ma Lourdes Bunyi, Veerendra Melagireppa Chadachan, Yook Chin Chia, Kazuomi Kario, Cheol-Ho Kim, Hung-Ju Lin, Noriko Matsushita, Sungha Park, Ebtehal Salman, Apichard Sukonthasarn, Jam Chin Tay, Hoang Anh Tien, Isha Tomar, Yuda Turana, Huynh Van Minh, Narsingh Verma, Gurpreet Singh Wander, Ji-Guang Wang, Yi Zhou, Yutaka Imai

**Affiliations:** 1 Cardiovascular Center and Divisions of Hospital Medicine and Cardiology, Department of Internal Medicine, National Taiwan University Hospital, No. 7, Zhong-Shan South Road, 100225 Taipei City, Taiwan, ROC; 2 Department of Hygiene and Public Health, Teikyo University School of Medicine, 2-11-1 Kaga, Itabashi-ku, Tokyo, 173-8605 Japan; 3 Dr. HB Calleja Heart and Vascular Institute, St. Luke’s Medical Center, 279 E. Rodriguez Sr. Avenue, Quezon City, 1102 Philippines; 4 Department of General Medicine, Tan Tock Seng Hospital, 11, Jalan Tan Tock Seng, 308433 Singapore; 5 Department of Medical Sciences, School of Medical and Life Sciences, Sunway University, 5 Jalan Universiti, Bandar Sunway, 47500 Selangor Darul Ehsan, Malaysia; 6 Department of Primary Care Medicine, Faculty of Medicine, University of Malaya, Lembah Pantai, 50910 Kuala Lumpur, Malaysia; 7 Division of Cardiovascular Medicine, Department of Medicine, Jichi Medical University School of Medicine, 3311-1, Yakushiji, Shimotsuke, Tochigi, 329-0498 Japan; 8 Department of Internal Medicine, Seoul National University College of Medicine, 103 Daehak-ro, Jongno-gu, Seoul, 03080 Republic of Korea; 9 Cardiovascular Center, Department of Internal Medicine, Seoul National University Bundang Hospital, 82 Gumi-ro 173 (baekchilsipsam) beo, Bundang-gu, Seongnam-si, Gyeonggi-do, 13620 Republic of Korea; 10 Asia Pacific Global Medical Affairs, Omron Healthcare Singapore, Pte. Ltd., 438B Alexandra Road #08-01/02, Alexandra TechnoPark, 119968 Singapore; 11 Yonsei University College of Medicine, Severance Cardiovascular Hospital, Division of Cardiology, 50-1 Yonsei Ro Seodaemungu Ludlow Faculty Building, Seoul, 03722 Republic of Korea; 12 Asia Pacific Global Medical Affairs, Omron Healthcare Co., Ltd., 53, Kunotsubo, Terado-cho, Muko, Kyoto, 617-0002 Japan; 13 Department of Medicine, Bangkok Hospital Chiang Mai, 88/8 Moo6, Tumbol Nong Pa Khrang, Amphur Muang Chiang Mai, Chiang Mai, 50000 Thailand; 14 Cardiovascular Department, Hue University of Medicine and Pharmacy, Hue university, Hue, 06 Ngo Quyen, Vinh Ninh District, Hue city, Thua Thien Hue province 52000 Vietnam; 15 Asia Pacific Global Medical Affairs, Omron Healthcare India Private Ltd., 6th Floor, B-Block, Sewa Tower, Plot No. 19, Sector-18, Udyog Vihar, Gurugram, Haryana 122008 India; 16 Department of Neurology, School of Medicine and Health Sciences, Atma Jaya Catholic University of Indonesia, Pluit Raya no 2, North Jakarta, 14440 Indonesia; 17 Department of Internal Medicine, Hue University of Medicine and Pharmacy, Hue University, 06 Ngo Quyen, Vinh Ninh District, Hue city, Thua Thien Hue province 52000 Vietnam; 18 Professor Department of Physiology, Officiating Head Department of Family Medicine, King George’s Medical University Lucknow, Lucknow, 226003 India; 19 Professor & Head of Cardiology, Dayanand Medical College & Hospital Unit Hero DMC Heart Institute, Ludhiana, 141001 Punjab India; 20 Department of Hypertension, The Shanghai Institute of Hypertension, Ruijin Hospital, Shanghai Jiao Tong University School of Medicine, Ruijin 2nd Road 197, Shanghai, 200025 China; 21 Tohoku Institute for Management of Blood Pressure, 13-18, Station Plaza Building, Futsukamachi, Aobaku, Sendai, Miyagi 980-0802 Japan

**Keywords:** Asia, Home blood pressure monitoring, Hypertension

## Abstract

Uncontrolled hypertension is a significant problem in many parts of Asia. Effective management is essential to reduce the burden of hypertension. Home blood pressure monitoring (HBPM) is a promising tool that can aid in the diagnosis and management of hypertension. Experts from 11 countries/regions in Asia conceptualized a large-scale survey to examine the current realities of HBPM. A cross-sectional survey was conducted among health care professionals from China, India, Indonesia, Japan, Malaysia, the Philippines, Singapore, South Korea, Taiwan, Thailand, and Vietnam between November 2019 and June 2021. Physicians’ responses were summarized using descriptive statistics. A total of 7945 physicians participated in the survey. Among all respondents, 50.3% and 33.5% viewed HBPM as highly recognized by physicians and patients in their country/region, respectively. Lack of understanding of HBPM and concern with the accuracy and reliability of HBPM devices were identified as key barriers to HBPM recognition. Nearly all physicians (95.9%) reported recommending HBPM to their patients; however, they reported less than 50% of their patients measured home blood pressure (HBP). Among physicians who recommended HBPM, only 22.4% and 54.1% cited HBP diagnostic threshold values and timing of taking antihypertensive drugs that were consistent with available guidelines, respectively. The survey reveals that the recognition of HBPM as a valuable tool to diagnose and manage hypertension is suboptimal in most parts of Asia. Despite high recommendation of HBPM to hypertensive patients by physicians, there are considerable discrepancies between guidelines recommendations and practice realities.

**Figure:**
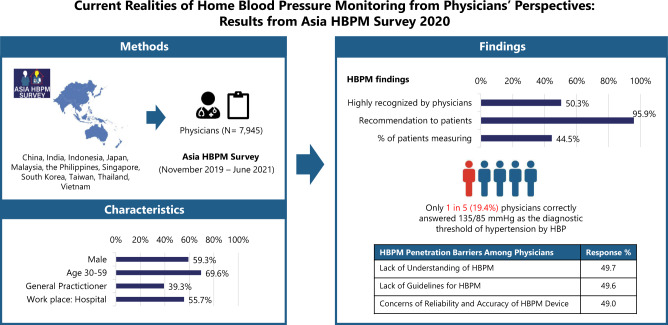
The recognition of HBPM as a valuable tool for the diagnosis and management of hypertension is suboptimal among both physicians and patients in Asia. A clear and consistent guidance for proper HBPM practice and use of validated and calibrated HBP monitors are among the top priorities to support the integration of HBPM into daily patient care. HBPM: home blood pressure monitoring, HBP: home blood pressure.

The recognition of HBPM as a valuable tool for the diagnosis and management of hypertension is suboptimal among both physicians and patients in Asia. A clear and consistent guidance for proper HBPM practice and use of validated and calibrated HBP monitors are among the top priorities to support the integration of HBPM into daily patient care. HBPM: home blood pressure monitoring, HBP: home blood pressure.

## Abstract (web summary)

The recognition of HBPM as a valuable tool for the diagnosis and management of hypertension is suboptimal among both physicians and patients in Asia. A clear and consistent guidance for proper HBPM practice and use of validated and calibrated HBP monitors are among the top priorities to support the integration of HBPM into daily patient care. HBPM: home blood pressure monitoring, HBP: home blood pressure.

## Introduction

As a continent with 60% of the world’s population, a rich cultural and ethnic diversity, as well as differences in socioeconomic developments and health care systems, Asia faces many challenges in the management of hypertension [1]. A 2019 worldwide survey with 104 million respondents noted that hypertension control and treatment rates varied widely across Asia [2]. South Korea and Taiwan were among the countries/regions with the highest hypertension control and treatment rates, whereas several countries/regions in central and south Asia have the lowest rates [2]. Overall, hypertension control remains suboptimal in Asia [2]. Uncontrolled hypertension remains a major contributor to cardiovascular disease (CVD) mortality and morbidity [3], and studies reported stronger association between CVD risk and hypertension in Asian compared with Western populations [4]. Among all CVD deaths in the region, it was estimated that up to 66% might be attributable to hypertension [5]. Therefore, effective management of hypertension is needed to reduce CVD mortality and morbidity in the region [2].

Accurate blood pressure (BP) measurement is essential for screening, diagnosis and management of hypertension [6]. Home blood pressure monitoring (HBPM) provides a reliable, convenient and cost-effective method for patients to self-monitor their BP at home [6, 7]. The integration of HBPM into hypertension practice is an important strategy to support better hypertension control. A growing body of evidence showed HBPM is a better predictor of CVD prognosis and hypertensive target organ damage than clinic blood pressure (CBP) [6–8], and even ambulatory BP monitoring [9]. HBPM is also helpful in identifying white-coat and masked hypertension which can be misdiagnosed using only CBP for hypertension diagnosis [6, 7]. Beyond diagnostic and prognostic values, HBPM offers patients to be actively involved in managing their BP, empowering them to take control of their health [6, 7]. Despite the wider community increasingly recognizing the importance of HBPM, explicit recommendations and proper guidance for accurate HBPM are lacking in many countries/regions in Asia [10].

To improve hypertension control in Asia, a group of 12 leading experts from ten Asian countries/regions (China, India, Indonesia, Japan, Malaysia, the Philippines, Singapore, South Korea, Taiwan, and Thailand) gathered in 2019 to share their perspectives on the situation of HBPM and challenges to HBPM usage [10]. The experts highlighted that HBPM usage was low in the region, especially among general practitioners (GPs) [10]. They revealed limited local guidance on HBPM in Asia except for China, Indonesia, and Japan, where local HBPM guidelines are available for physicians [10]. The experts recommended conducting more local HBPM research and developing local HBPM guidelines as a foundational step for increasing HBPM recognition and usage [10].

Following the summit in 2019, the experts and a few others, representing 11 countries/regions in Asia (with the addition of Vietnam), conceptualized a large-scale survey. Taiwan and South Korea also published their local HBPM guidelines in 2020 and 2021, respectively [11, 12]. The survey sought to examine HBPM recognition, usage, and understanding, as well as identify factors hindering its recognition from physicians’ perspectives. This paper summarizes the survey results to provide a landscape of the current realities of HBPM in Asia.

Point of View

- Clinical relevance
  Despite high recommendation of home blood pressure monitoring to hypertensive patients by physicians in Asia, there are considerable discrepancies between guidelines recommendations and practice realities.
- Future direction
  A clear and consistent guidance for proper home blood pressure monitoring practice and use of validated and calibrated home blood pressure monitors are among the top priorities to support the integration of home blood pressure monitoring into daily patient care.
- Consideration for the Asian population
  To improve the adoption of home blood pressure monitoring in Asia, recognition of the unique challenges in each country/region and engagement of multiple stakeholders are essential.

## Methods

This cross-sectional survey was conducted among health care professionals (mainly physicians) from 11 countries/regions in Asia (China, India, Indonesia, Japan, Malaysia, the Philippines, Singapore, South Korea, Taiwan, Thailand, and Vietnam) between November 2019 and June 2021. The survey was approved by the Institutional Review Board (IRB) and granted exemption from full review. Health care professionals who consented to participate in the survey completed the questionnaire.

### Questionnaire design and data collection

The questionnaire consisted of 24 questions covering HBPM recognition and usage, perceived barriers to HBPM recognition, HBP devices validation, timing and frequency of HBPM, HBPM instructions, and awareness of diagnostic threshold values (Supplementary Table 1). The questionnaire was adapted based on the published study by Obara et al. [13], and modified by the experts involved in this study. Permission to use questions from the original survey was obtained. Some countries/regions included additional questions or answer choices to understand their respective country’s/region’s situation better. The questionnaire was translated to a few local languages as required. The questionnaire soft or hard copies were distributed locally to health care professionals through public universities, medical societies and conferences, and hypertension educational seminars. Responses to the following aspects of HBPM are reported in the present paper: HBPM recognition and usage; perceived barriers towards HBPM recognition; instructions to measure HBPM; and awareness of diagnostic threshold values.

### Statistical analyses

All analyses were performed using JMP statistical software, version 15.2.1 (SAS Institute, Cary, NC, US). The analysis included only physicians and excluded other health care professionals. Participants with missing data from the characteristic questions of the survey (questions 1–5) were excluded. Physicians’ responses were summarized for the overall sample, as well as by country using descriptive statistics expressed as percentage only. Cross-tabulations were used to examine the relationship between physicians’ recommendations for HBPM and their knowledge of HBP diagnostic thresholds or instructions for HBPM.

## Results

A total of 7945 physicians participated in the survey (Table 1). The majority were male (59.3%) and aged 30–59 years (69.6%). Among all respondents, 50.3% practiced internal medicine, 39.3% were GPs, and 9.8% were from other specialties. Of those from internal medicine, 31.7% were internist, 30.7% were cardiologist, 7.1% were nephrologist, 4.8% were neurologist. Most of the respondents worked in hospitals (55.7%) or clinics (41.5%). There was a notable difference in the proportion of physicians who worked in hospitals, with the highest in China (95.7%) and lowest in South Korea (5.5%). More than half of the physicians (52.1%) managed at least 30 hypertensive patients weekly.

**Table 1:** Overall participants’ characteristics of Asia HBPM survey 2020

Characteristics	*N* = 7945
Gender	
Male	59.3
Female	38.4
Other	0.1
Age	
20–29 years	11.9
30–39 years	26.5
40–49 years	22.2
50–59 years	20.9
60–69 years	12.9
70–79 years	4.3
80 years or more	0.8
Specialty	
General Practitioner (GP)	39.3
Internal medicine	50.3
Others	9.8
Internal medicine specialty	
Cardiologist	30.7
Internist	31.7
Nephrologist	7.1
Neurologist	4.8
Others	13.0
Workplace	
Hospital	55.7
Clinic	41.5
Others	1.9
Number of hypertension patients managed/week	
None	1.3
less than 10	10.8
10–19	14.1
20–29	13.8
30–39	10.4
40–49	4.5
50–99	18.2
100–200	16.0
more than 200	3.0
Other	2.1

Values are presented as percentages only

### HBPM recognition and usage

**Fig. 1:**
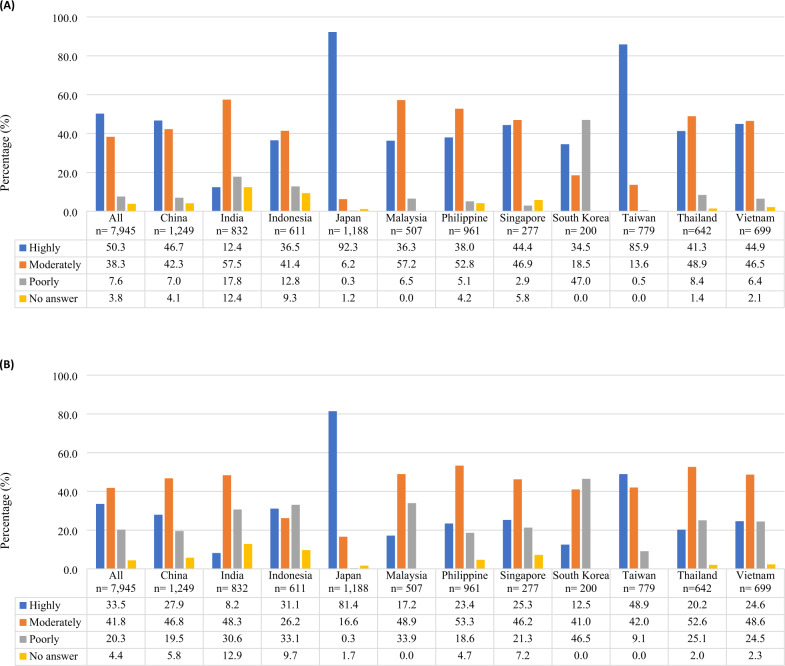
HBPM recognition in Asia (**A**) among physicians upon answering the question [11) Do you think that the significance of HBPM is well recognized by physicians in your country?] (**B**) among patients upon answering the question [12) Do you think that the significance of HBPM is well recognized by patients with hypertension in your country?]. Results for patients are based on physicians’ perspective. HBPM home blood pressure monitoring

HBPM recognition among physicians and patients reported by the survey respondents are summarized in Fig. 1. The survey revealed considerable differences in HBPM recognition between the countries/regions. Overall, only half of the survey respondents (50.3%) viewed HBPM as highly recognized by physicians in their country/region (Fig. 1A). Less than 50% of the physicians surveyed (12.4–46.7%) considered HBPM as highly recognized, except for Japan (92.3%) and Taiwan (85.9%). From physicians’ perspectives, HBPM recognition was even lower among patients, with only 33.5% of physicians reported HBPM as highly recognized by patients (Fig. 1B). Less than half of the physicians in the majority of the countries/regions viewed HBPM as highly recognized by patients (8.2–48.9%), except for Japan (81.4%).

**Fig. 2:**
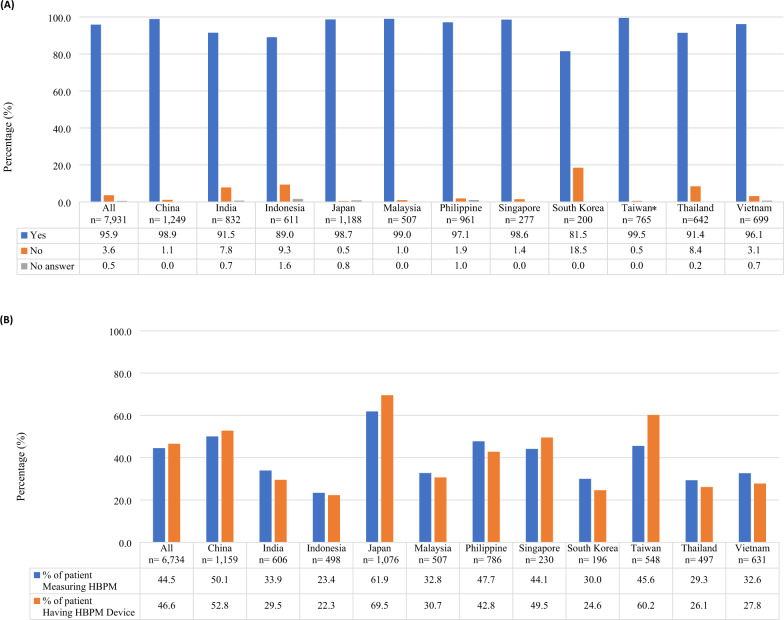
HBPM usage in Asia (**A**) among physicians upon answering the question [8) Do you recommend HBPM to your patients with hypertension?] **B** among patients upon answering the question [9) What percentage of your patients with hypertension measures their own BP (HBP)? and 10) What percentage of your patients with hypertension have HBPM devices?]. *Exclude “impossible to select either” option. Percentage of patients calculated based on number of doctors who responded to question 9 and 10. Results for patients are based on physicians’ perspective. HBP home blood pressure; HBPM home blood pressure monitoring

HBPM recommendation by physicians and perceived usage among patients are summarized in Fig. 2. Around 96% of physicians indicated they recommended HBPM to their hypertensive patients, with slightly lower proportion in South Korea (81.5%) and Indonesia (89.0%) than the rest of the countries/regions (>90.0%) (Fig. 2A). Despite the high percentage recommending HBPM, respondents indicated less than 50% of their patients owned HBPM devices and measured HBP (Fig. 2B). Regarding HBPM ownership, Japan (69.5%) and Taiwan (60.2%) had the highest percentage of patients owning a HBPM device, whereas Indonesia (22.3%) and South Korea (24.6%) recorded the lowest numbers. Only Japan (61.9%) and China (50.1%) recorded more than half of their patients measured HBP, whereas Indonesia (23.4%) and Thailand (29.3%) noted the lowest proportion.

### Perceived barriers to HBPM recognition

**Fig. 3:**
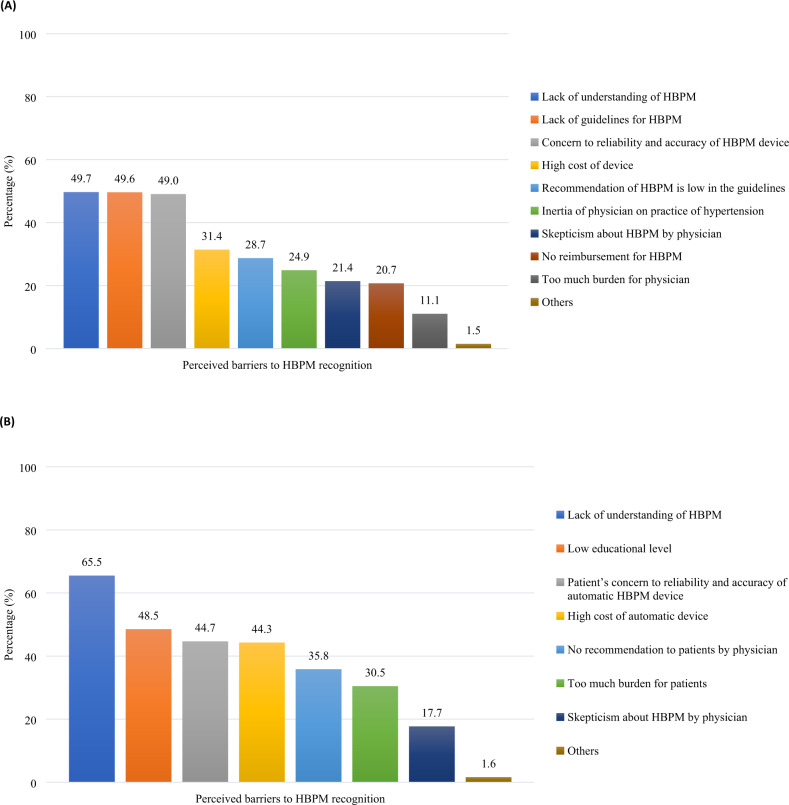
Perceived barriers to HBPM moderate/low recognition in Asia (**A**) by physicians (*n* = 3647) upon answering moderately or poorly to question [11) Do you think that the significance of HBPM is well recognized by physician in your country?] (**B**) by patients (*n* = 4931) upon answering moderately or poorly to question [12) Do you think that significance of HBPM is well recognized by patients with hypertension in your country?]. Results for patients are based on physicians’ perspective. HBPM home blood pressure monitoring

Top barriers to HBPM recognition identified by physicians included lack of HBPM understanding (49.7%), lack of HBPM guidelines (49.6%), concerns with HBPM reliability and accuracy (49.0%), and high cost of device (31.4%) (Fig. 3A). Notably, a fairly high proportion indicated a lack of guidelines as a major barrier even in China (52.2%) and Indonesia (47.1%), where local HBPM guidelines/consensus have been published at the time of the survey.

Similarly, physicians cited lack of HBPM understanding (65.5%), concerns regarding reliability and accuracy (44.7%), and high cost of device (44.3%) as key reasons hindering HBPM recognition by patients (Fig. 3B). Nearly half reported low educational level as a reason for low or moderate recognition of HBPM in patients, with at least 50% noted in Malaysia (63.1%), Taiwan (58.5%), Indonesia (56.1%), India (54.5%), China (52.5%), and Vietnam (50.1%).

### Awareness of diagnostic thresholds for HBP and CBP measurements

**Fig. 4:**
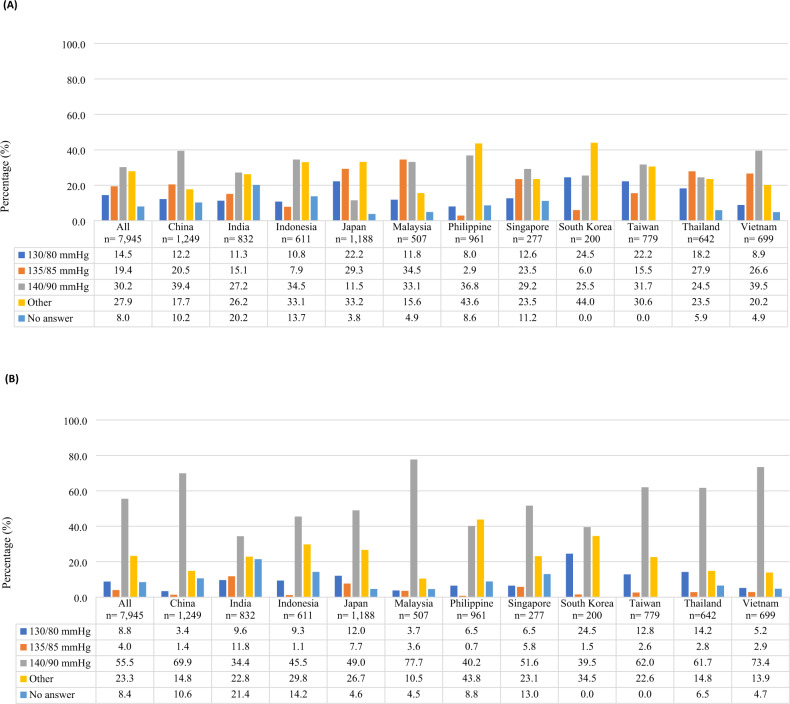
Awareness of hypertension reference values among physicians (**A**) by HBP upon answering the question [23) Please indicate the reference values of hypertension for HBP with your own view.] (**B**) by CBP upon answering the question [24) Please indicate the reference values of hypertension for CBP with your own view]. CBP clinic blood pressure; HBP, home blood pressure

The overall distribution of perceived diagnostic thresholds for HBP and CBP measurements are summarized in Supplementary Figs. 1and 2. Among all physicians, only 19.4% and 55.5% identified 135/85 mmHg and 140/90 mmHg as the diagnostic values for HBP and CBP measurements, respectively (Fig. 4A, B, respectively). Notably, 30.2% of physicians indicated 140/90 mmHg as the diagnostic threshold for HBPM. Among the countries/regions, the Philippines (2.9%), South Korea (6.0%), and Indonesia (7.9%) had the lowest proportion of physicians who selected 135/85 mmHg for HBPM compared with the rest of the countries (15.1–34.5%). Further analysis showed that of those who indicated they recommended HBPM to their patients, only 22.4% cited diagnostic threshold values for HBPM that were consistent with their country/region’s guidelines (135/85 mmHg) (Supplementary Table 2).

### Instructions for HBPM

**Fig. 5:**
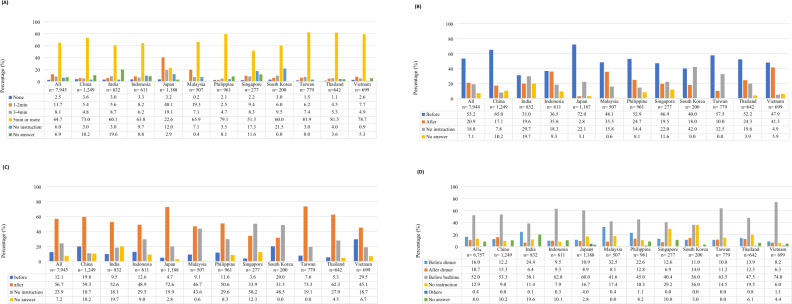
Instructions to measure HBPM given by physicians. upon answering question [20) Please select your instruction to your patients regarding HBPM in the morning] (**A**) Time of rest before measurement. **B** HBPM relative to taking antihypertensive medications. **C** Micturition. **D** Upon answering timing of measurement in the evening to question [21) Please select your instruction to your patients regarding HBPM in the evening.]. *‘All’ data does not include Japan. †Multiple answers and ‘before/after bathing’ option not shown – before bathing 12.8%, after bathing 3.5%. HBPM home blood pressure monitoring

Several instructions regarding HBPM were evaluated (i) time of rest before measurement, (ii) timing of taking antihypertensive drug (iii), timing of micturition, and (iv) timing of HBPM in the evening. In general, there were considerable variations in physicians’ instructions given to patients (Fig. 5).

The majority of physicians (64.7%) instructed patients to rest for five minutes or more before measuring HBPM, whereas 11.7% taught patients to measure after waiting for 1–2 min (Fig. 5A). Around 6.0% of physicians did not provide instructions of resting time with the highest proportion in South Korea (21.5%) and Singapore (17.3%).

The proportion of physicians who recommended measuring HBP prior to taking antihypertensive medications was 53.2% (Fig. 5B), with Japan (72.0%) and China (65.0%) having the highest proportion. Around 21% recommended measuring HBP after taking antihypertensive medications and 18.8% did not provide any instructions. Of the physicians who recommended HBPM to their patients, only 54.1% provided instructions that were consistent with their respective local guidelines (measure HBP before taking antihypertensive medications) (Supplementary Table 3).

Regarding micturition, 56.7% recommended measuring HBP after micturition, with the highest proportion in Taiwan (73.3%) and Japan (72.6%), and lowest in South Korea (31.5%) and Singapore (33.9%) (Fig. 5C). Overall, nearly one quarter did not provide any instructions relating to micturition. Over 10% instructed patients to measure HBP before micturition.

On the timing of HBPM in the evening, most physicians recommended measuring HBPM before bedtime (52.0%), with the highest proportion in Vietnam (74.0%) and lowest in South Korea (36.0%) and India (38.1%) (Fig. 5D). Less than one-fifth recommended patients to measure HBP before dinner (16.0%), after dinner (10.7%), or did not provide any instructions (12.9%).

## Discussion

To our knowledge, this is the first large-scale survey involving multiple countries/regions in Asia that assessed physicians’ perspectives on the current realities of HBPM in the region. This survey expanded on findings from a previous panel discussion on the status of HBPM in Asia and key factors hindering its usage [10]. Of the participating countries/regions, Thailand has recently published their individual country’s data [14]. Although nearly all physicians indicated they recommended HBPM to their patients, less than half of their patients owned a HBPM device or measured their HBP. Furthermore, a substantial proportion of physicians did not provide diagnostic thresholds or instructions for HBPM that were consistent with the recommendations in available guidelines. Taken together, these findings suggest that although physicians recommend HBPM to their patients, many still do not regard HBPM as an integral part of hypertension management.

In the present survey, a high proportion of physicians indicated HBPM as not well recognized by both physicians and patients in most of the countries/regions in Asia (except Japan). The physicians cited lack of HBPM understanding and guidelines as key barriers to HBPM recognition by physicians. These findings are in line with the landscape at the time of the survey where the majority of the Asian countries/regions (India, Malaysia, the Philippines, Singapore, South Korea, Taiwan, Thailand, and Vietnam) did not have local HBPM guidelines. Furthermore, even in China and Indonesia, where local HBPM guidelines/consensus are available at the time of the survey, a sizeable proportion of physicians cited the lack of HBPM guidelines as a reason for low or moderate recognition. This highlights a lack of awareness of local HBPM guidelines/consensus among physicians in these countries/regions. In addition, physicians identified concerns with the accuracy and reliability of HBP devices as a key factor hindering the recognition of HBPM by physicians and patients. This could be attributed to the lack of instructions on device calibration in the local guidelines of most countries/regions, except for China, Indonesia, and Japan [15–17]. As with all devices, it is important to ensure HBP monitors are calibrated and validated. Local guidelines should advocate using calibrated HBPM devices and provide specific instructions on calibration to support accurate measurements [18].

The translation of guideline recommendations to clinical practice is a complex yet important process. Our results showed that, while a high proportion of physicians recommended HBPM to their patients, discrepancies between HBPM recommendations in guidelines and practice realities were prevalent. For instance, all of the countries/regions surveyed, except for the Philippines, recommended 135/85 mmHg as the diagnostic threshold for hypertension by HBP in general population in their guidelines (Supplementary Table 4) [15–17, 19–26]. However, overall, only one-fifth of the physicians cited 135/85 mmHg as the diagnostic threshold for HBPM. Further analysis showed a similar low proportion (22.4%) among physicians who indicated they recommended HBPM to their patients. Although most of the countries/regions recommended measuring HBPM before taking antihypertensive medications or after micturition in their guidelines (Supplementary Table 4) [15–17, 19–26], only slightly more than half of the physicians provided instructions that were consistent with guidelines recommendations. These findings were corroborated by local HBPM surveys of physicians in Indonesia, Japan, and Singapore which revealed that at least 94% recommended HBPM to hypertensive patients, however, a substantial proportion of physicians gave instructions that were inconsistent with guidelines recommendations for HBPM [13, 27, 28] Taken together, these findings suggest that even when HBPM is conceptually recognized by physicians and recommended to patients, there is still suboptimal translation of guideline recommendations to actual clinical practice across the participating countries.

The observed suboptimal translation of guideline recommendations to practice realities may be attributed to several factors. First, there may be insufficient education on HBPM resulting in limited awareness of available guidelines among physicians. A study in Singapore highlighted difficulties that GPs faced in familiarizing themselves with multiple guidelines for different conditions they are treating [29] Second, the lack of clear and simple HBPM recommendations in guidelines might be another factor. For instance, the Philippines and Singapore do not provide instructions on how to measure HBP, and the Philippines did not provide the HBP diagnostic threshold value for hypertension in their hypertension guidelines. Next, a lack of agreement with guidelines recommendations could arise as physicians are not convinced with the level of available evidence. For instance, recommendations on HBPM are based on the consensus of experts in the field and the diagnostic threshold value of 135/85 mmHg for HBPM is mainly derived from a meta-analysis of prospective studies done by IDHOCO (International Database of HOme blood pressure in relation to Cardiovascular Outcome) [30] Another possible reason may be that physicians have limited time during consultations to guide their patients in taking proper HBPM.

Our survey showed that HBPM was not well recognized by both physicians and patients in most of the countries/regions in Asia. Less than half of the patients owned HBP devices and measured HBP. This is reminiscent of observations from two small studies in Asia which reported only a quarter to about half of hypertensive patients possessing a HBP monitor [31, 32]. The observed suboptimal recognition of HBPM and low HBP monitor ownership and HBP measure rate may partly explain suboptimal hypertension control noted in many parts of Asia [2].

Integrating HBPM into routine patient care is important to support optimal management of hypertension. The experts proposed several future actions to improve the current situation of HBPM. It would benefit all countries to have local HBPM guidelines/consensus and develop Pan-Asian HBPM guidelines which should include clear and practical guidance for proper and accurate HBPM. Mnemonics like the “722” protocol, which summarizes key aspects of HBPM (average of 7 day measurements, 2 occasions [morning and evening] a day, and 2 measurements on one occasion) has been advocated by the Taiwan Hypertension Society and could facilitate the implementation of HBPM [12]. Additionally, standardization of HBPM practices is indispensable for comparability of HBPM and increase its clinical significance between countries/regions. HBPM recommendations and educational resources should be disseminated through ongoing education efforts such as seminars, webinars, or training sessions and providing user-friendly and easy-to-follow visual tools and resources to improve HBPM recognition for the physicians, general public and patients. Local hypertension societies should take the lead to endorse HBPM for hypertension practice and advocate the use of certified devices. Government authorities should also strive to improve access to reliable HBPM devices by dissemination of validated and inexpensive HBPM devices, ensuring certification of HBPM devices by regulatory authorities, and implementing reimbursement programs for HBP monitors, particularly in low-income countries. For instance, Thailand is working towards providing reimbursement for HBP monitors to promote HBPM usage.

While this survey had a large sample size and included several countries/regions in Asia, there are some limitations. First, most respondents were either GPs or physicians from internal medicine specialties, hence the results may not be generalizable to other physicians. Nonetheless, it should be noted that our respondents represent the majority of physicians who manage hypertensive patients. Next, our survey collected responses from physicians, hence it could not capture patients’ perspectives directly. It would be important to gather patients’ perspectives on HBPM in future work. Furthermore, differences in health care system and specialties between countries/regions could influence physicians’ responses. In addition, the survey data were collected from physicians through professional societies, public universities, hypertension educational seminars, and medical conferences, rather than via national representative sampling. These could contribute to selection bias. Besides, there were minor differences in the survey design for some countries, which may limit the comparability between each country/region. For example, the survey in Japan had more options for the question on evening HBPM, and respondents could select more than one answer. Therefore, caution should be taken when interpreting and comparing the results between countries. Lastly, some of the survey findings may be influenced by social desirability bias due to the tendency for respondents to select answers that appear more socially acceptable. Nonetheless, this survey provides valuable insights regarding the current realities of HBPM in 11 countries/regions in Asia.

### Perspective of Asia

This is the largest survey involving multiple countries/regions in Asia that assessed physicians’ perspectives on the current status of HBPM in hypertension management. The survey results showed that although physicians recommended HBPM to their patients, many still did not consider HBPM as an indispensable part of hypertension management. Lack of understanding of HBPM and concern with the accuracy and reliability of HBPM devices were identified as key barriers to HBPM recognition. To improve the adoption of HBPM in Asia, recognition of the barriers in each country/region is the essential first step.

In conclusion, this large-scale survey on physicians from 11 countries/regions in Asia shows that the recognition of HBPM as a valuable tool for the diagnosis and management of hypertension is suboptimal among both physicians and patients in most parts of Asia. Despite high recommendation of HBPM to hypertensive patients by physicians, there are considerable discrepancies between guideline recommendations and practice realities. These findings reveal that much work still needs to be done to improve the use of HBPM in the region. This would require recognition of the unique challenges in each country/region and engagement of multiple stakeholders to promote HBPM recognition and usage. A clear and consistent guidance for proper HBPM practice and use of validated and calibrated HBP monitors are among the top priorities to support the integration of HBPM into daily patient care.

## Supplementary Information

Supplementary information
