# Supplementary material for: Current realities of home blood pressure monitoring from physicians’ perspectives: results from Asia HBPM survey 2020
Source: Hypertens Res. 2023 Apr 11;46(7):1638–49. doi: 10.1038/s41440-023-01259-1 (PMC10319632; doi:10.1038/s41440-023-01259-1)
Supplement: Supplementary file 1 — Supplementary information [file 41440_2023_1259_MOESM1_ESM.docx]

# Supplementary Table 1. Asia HBPM Survey Questionnaire

| **1) Occupation** 1. Doctor  2. Pharmacist  3. Nurse  4. Public health nurse  5. Others |
| --- |
| **2) Gender** 1. Male  2. Female  3. Others |
| **3) Age** 1. 20–29 years 2. 30–39 years 3. 40–49 years 4. 50–59 years  5. 60–69 years 6. 70–79 years 7. 80 years or more |
| **4) Specialty** 1. Internal medicine 2. General Practitioner (GP) 3. Others (Specialty: ) |
| **(A) If you selected “Internal medicine” please answer** 1. Cardiologist 2. Neurologist 3. Nephrologist  4. Internist 5. Others ( ) |
| **5) Workplace** 1. Hospital 2. Clinic 3. Others　( ) |
| **6) In your practice, how many patients with hypertension do you manage weekly?**  (≒ 　 ) number of patients / weekly |
| **7) Which BP measurement is the most important for you to decide on treatment of patients with hypertension?** 1. CBP 2. HBP 3. ABP |
| **8) Do you recommend HBP measurement to your patients with hypertension?** 1. Yes 2. No |
| 1. **If you selected “Yes”, please answer which type of patients do you recommend HBP measurements? (you may choose more than one answer)** 1. All patients 2. Newly diagnosed patients with hypertension only 3. Already known hypertension patients only 4. Patients on drug treatment 5. Patients on non-pharmacological treatment 6. Patients with low drug compliance / adherence 7. Others |
| **(B) If you selected “No”, please provide possible reasons (you may choose more than one answer)** 1. Lack of guidelines for HBPM  2. Lack of understanding of HBPM 3. Recommendation of HBPM is low in the guidelines 4. No reimbursement for HBPM  5. Skepticism about HBPM 6. Too much burden for physician 7. Concern on reliability and accuracy of HBPM device 8. High cost of device 9. Inertia of physician on practice of hypertension 10. Others (Free answer: ) |
| **9) What percentage of your patients with hypertension measures their own BP(HBP)?**  (≒ 　　　 )% |
| **10) What percentage of your patients with hypertension have HBPM devices?**  (≒ )% |
| 1. **Which type of HBPM device is mainly used among your patients?** 1. Automatic electrical device 2. Aneroid device 3. Mercury device |
| 1. **Which type of HBPM device do you recommend to your patients?** 1. Automatic electrical device 2. Aneroid device 3. Mercury device |
| 1. **Which automatic device do you recommend to your patients with hypertension to measure their HBP?** 1. Upper-arm-cuff device 2. Wrist device 3. Finger device 4. None specified |
| **11) Do you think that the significance of HBPM is well recognized by physician in your country?** 1. Highly  2. Moderately 3. Poorly |
| **(A) If you selected “moderately or poorly”, please provide possible reasons (you may choose more than one answer)** 1. Lack of guidelines for HBPM  2. Lack of understanding of HBPM 3. Recommendation of HBPM is low in the guidelines 4. No reimbursement for HBPM  5. Skepticism about HBPM by physician 6. Too much burden for physician 7. Concern to reliability and accuracy of HBPM device 8. High cost of device 9. Inertia of physician on practice of hypertension 10. Others (Free answer: ) |
| **12) Do you think that significance of HBPM is well recognized by patients with hypertension in your country?** 1. Highly  2. Moderately 3. Poorly |
| **(A) If you selected “moderately or poorly”, please provide possible reasons (you may choose more than one answer)** 1. Lack of understanding of HBPM 2. No recommendation to patients by physician 3. Skepticism about HBPM by physician 4. Too much burden for patients  5. Patient’s concern to reliability and accuracy of automatic HBPM device 6. High cost of automatic device 7. Low educational level 8. Others (Free answer: ) |
| **13) In your opinion, what are the benefits of HBPM? (you may choose more than one answer)**  1. Hypertension management based on HBP 2. Diagnosis of white-coat hypertension 3. Diagnosis of masked hypertension 4. Diagnosis of resistant hypertension 5. Awareness of hypertension by patients 6. Improvement of drug compliance 7. Evaluation of efficacy of antihypertensive drugs 8. Detection of duration of the action of the anti-hypertensive drug effects (e.g. morning effect versus the evening effect ratio) 9. Evaluation of patients’ CVD risk 10. Evaluation of BP variation 11. Diagnosis of hypotension  12. Others (Free answer: ) |
| **14) Generally, what do you think about the accuracy and reliability of automatic HBPM device?** 1. Excellent 2. Good 3. Fair 4. Poor 5. Undefined |
| **15) Do you regularly validate the accuracy of HBPM device by comparing with auscultation?** 1. Yes  2. No |
| **16) How long should HBP be measured?** 1. For a few days 2. For a week 3. For a month 4. For a several months 5. For a year 6. For several years 7. For a lifelong |
| **17) How many days do you recommend to your patients to measure HBP weekly?** 1. One day 2. Two days 3. Three days 4. Four days 5. Five days 6. Six days 7. Seven days |
| **18) On each occasion, which HBP do you instruct your patients to record?** 1. First measurement 2. Second measurement 3. Third measurement  4. All measurements 5. Mean of measurements 6. No instruction 7. Others (Free answer: ) |
| **19) How do you evaluate the measurements obtained by the HBP measurement?** 1. Mean value of measurements 2. Individual value of measurement 3. Both  4. Others (Free answer: ) |
| **20) Please select your instruction to your patients regarding HBPM in the morning.** |
| 1. **Timing of measurement after waking up** 1. Just after 2. Within 30min 3. Within an hour 4. No instruction |
| 1. **Micturition** 1. Before 2. After 3. No instruction |
| 1. **Body position** 1. Sitting position 2. Recumbent position 3. No instruction |
| 1. **Time of rest before measurement** 1. None 2. 1–2min 3. 3–4min 4. 5min or more  5. No instruction |
| 1. **Taking antihypertensive drug** 1. Before 2. After 3. No instruction |
| 1. **Breakfast** 1. Before 2. After 3. No instruction |
| **21) Please select your instruction to your patients regarding HBPM in the evening.**  1. Before dinner 2. After dinner 3. Before bedtime 4. No instruction 5. Others (Free answer: ) |
| **22) Which HBP measurement help you decide on the treatment of patients with hypertension?** 1. Morning HBP 2. Evening HBP  3. Morning/Evening equally  4. Others (Free answer: ) |
| **23) Please indicate the reference values of hypertension for HBP with your own view.** Systolic ( ) mmHg Diastolic ( ) mmHg |
| **24) Please indicate the reference values of hypertension for CBP with your own view.** Systolic ( ) mmHg Diastolic ( ) mmHg |

The survey questionnaire is a modified version of the survey used in the published paper Obara et al [13]. Permission to use questions from the original survey was obtained from the authors.

**BP**: blood pressure, **ABP**: ambulatory blood pressure, **HBP**: home blood pressure, **CBP**: casual-clinic blood pressure, **CVD**: cardiovascular disease, **HBPM**: home blood pressure monitoring

# Supplementary Figure 1. Overall distribution of physicians' responses on the reference values of hypertension for HBP. a) Systolic HBP (N=7,279) b) Diastolic HBP (N=7,277)

a)

b)

# Supplementary Figure 2. Overall distribution of physicians' responses on the reference values of hypertension for CBP. a) Systolic CBP (N=7,253) b) Diastolic CBP (N=7,254)

a)

b)

# Supplementary Table 2. Physician’s recommendations for HBPM and their knowledge of HBP diagnostic threshold for hypertension

|  | | **All** | **China** | **India** | **Indonesia** | **Japan** | **Malaysia** | **Singapore** | **South Korea** | **Taiwan** | **Thailand** | **Vietnam** |
| --- | --- | --- | --- | --- | --- | --- | --- | --- | --- | --- | --- | --- |
|  |  | **6970** | **1249** | **832** | **611** | **1188** | **507** | **277** | **200** | **765** | **642** | **699** |
|  |  | **Q8. Do you recommend HBP measurement to your patient with hypertension?** | | | | | | | | | | |
|  |  | Yes | Yes | Yes | Yes | Yes | Yes | Yes | Yes | Yes | Yes | Yes |
|  |  | 6670 | 1235 | 761 | 544 | 1172 | 502 | 273 | 163 | 761 | 587 | 672 |
| **Q23. Please indicate the reference values of hypertension for HBP with your own view** | Consistent | 22.4% | 20.6% | 16.3% | 7.9% | 29.5% | 34.7% | 23.8% | 7.4% | 15.9% | 29.8% | 26.5% |
|  | Inconsistent | 70.7% | 69.3% | 69.1% | 79.8% | 67.2% | 60.4% | 65.2% | 92.6% | 84.1% | 64.1% | 68.8% |
|  | No answer | 7.0% | 10.0% | 14.6% | 12.3% | 3.3% | 5.0% | 11.0% | 0.0% | 0.0% | 6.1% | 4.8% |

Consistent: In agreement with HBP reference value in country’s hypertension/HBP guidelines (135/85 mm Hg), Inconsistent: other answers.

# Supplementary Table 3. Physician’s recommendations for HBPM and their knowledge of the timing to take antihypertensive drugs during morning HBPM

|  | | **All** | **China** | **India** | **Indonesia** | **Japan** | **Malaysia** | **South Korea** | **Thailand** | **Vietnam** |  |
| --- | --- | --- | --- | --- | --- | --- | --- | --- | --- | --- | --- |
|  |  | **5927** | **1249** | **832** | **611** | **1187** | **507** | **200** | **642** | **699** |  |
|  |  | **Do you recommend HBP measurement to your patient with hypertension?** | | | | | | | | | |
|  |  | Yes | Yes | Yes | Yes | Yes | Yes | Yes | Yes | Yes |  |
|  |  | 5635 | 1235 | 761 | 544 | 1171 | 502 | 163 | 587 | 672 |  |
| **Q20e. Taking antihypertensive drug** | Consistent | 54.1% | 65.4% | 31.3% | 38.4% | 72.7% | 48.0% | 42.3% | 53.8% | 47.3% |  |
|  | Inconsistent | 21.5% | 16.8% | 20.2% | 35.8% | 2.7% | 35.5% | 15.3% | 23.0% | 42.1% |  |
|  | No instructions | 17.3% | 7.8% | 30.5% | 17.1% | 21.9% | 15.9% | 42.3% | 19.3% | 4.9% |  |
|  | No answer | 7.1% | 10.0% | 18.0% | 8.6% | 2.6% | 0.6% | 0.0% | 3.9% | 5.7% |  |

Consistent: in agreement with HBPM instructions on taking antihypertension drug in country’s hypertension/HBP guidelines (before taking medicine), Inconsistent: after taking medicine.

# Supplementary Table 4. Guideline recommendations at the time of survey.

| **Country/region** | **HBPM/HT**  **guidelines** | **HBPM threshold (mm Hg)** | **Morning measurement instructions** | | | **Timing of measurement in the evening** |
| --- | --- | --- | --- | --- | --- | --- |
|  |  |  | **Micturition** | **Time of rest before measurement** | **Antihypertensive drug** |  |
| **China** | HBPM: 2019 [17] | 135/85 | After | 5 mins | Before | After dinner, before bedtime |
| **India** | HT: 2019 [22] | 135/85 | - | 5 mins | Before | - |
| **Indonesia** | HBPM: 2019 [16] | 135/85 | After | 2 mins | Before | Before bedtime |
| **Japan** | HBPM: 2012 [15] | 135/85 | After | 1-2 mins | Before | Before dinner |
| **Malaysia** | HT: 2018 [23] | 135/85 | - | 1 min | Before | Before dinner |
| **Philippines** | HT: 2020 [19] | 135/85 | - | - | - | - |
| **Singapore** | HT: 2017 [20] | 135/85 | - | - | - | - |
| **South Korea** | HT: 2018 [26] | 135/85 | After | 1-2 mins | Before | Before bedtime |
| **Taiwan** | HT: 2015 [25] | 135/85 | After | 5 mins | - | - |
| **Thailand** | HT: 2019 [21] | 135/85 | After | 2 mins | Before | Before bedtime |
| **Vietnam** | HT: 2018 [24] | 135/85 | - | 5 mins | Before | - |
